# Kinetics of Orthostatic Blood Pressure in Primary Hypertension

**Published:** 2014-09-01

**Authors:** Fatima Zahra Milouk, Mustapha El Bakkali, Leslie Coghlan, Amal Lachhab, Souad Aboudrar, Halima Benjelloun

**Affiliations:** 1 Unit of Cardiology A, Ibn Sina University Hospital, Rabat 10000, Morocco; 2 Autonomic Nervous System Team (ERSNA), Faculty of Medicine and Pharmacy, University Mohammed V Souissi, Rabat 6203, Morocco; 3 Physiology of Exercise Team (LPE), Faculty of Medicine and Pharmacy, University Mohammed V Souissi, Rabat 6203, Morocco; 4 School of Science and Engineering, AL Akhawayn University, Ifrane, Morocco; 5 Research Center for Clinical Epidemiology and Therapeutic Trials (CRECET), Faculty of Medicine and Pharmacy, University Mohammed V Souissi, Rabat 6203, Morocco

**Keywords:** Primary Hypertension, Autonomic Nervous System, Orthostatic Hypotension

## Abstract

**Background:**

Primary Hypertension (HT) is the most prevalent cardiovascular disorder worldwide and is accompanied by significant morbidity and mortality.

**Objectives:**

The present study aimed to investigate the kinetics of orthostatic Blood Pressure (BP) in primary hypertensive patients during the change from supine position to standing position as well as during the standing position using the Orthostatic Test (OT).

**Patients and Methods:**

This prospective study included a group of 107 primary hypertensive patients (mean age: 55.82 ± 11.35 years, ranging from 39 to 80 years). Orthostatic systolic BP (Ortho SBP) was recorded for 10 minutes at the rhythm of 3 measurements per minute and was compared to the values of supine systolic preorthostatic (Preortho SBP). According to the changes in Ortho SBP, three subgroups of primary hypertensive patients were selected as follows: Subgroup A: Ortho SBP was higher than mean Preortho SBP by 10 mmHg or more. Subgroup B: Ortho SBP was lower than mean Preortho SBP by 20 mmHg or more. Subgroup C: -20 mmHg < (Ortho SBP - Preortho SBP) < + 10 mmHg.

The kinetics of each group was then recorded.

**Results:**

In this study, the prevalence of subgroups A, B, and C was 27.1%, 15.9%, and 57.0%, respectively. In subgroup A, the adrenergic peripheral sympathetic alpha response was 20% during the OT.

**Conclusions:**

Hypertensives with very similar supine SBP behavior could exhibit widely different Ortho SBP. Thus, careful and effective treatment of hypertensives requires careful consideration and assessment of orthostatic BP.

## 1. Background

Arterial Hypertension (HT) is defined as Systolic Blood Pressure (SBP) ≥ 140 mmHg and/or Diastolic Blood Pressure (DBP) ≥ 90 mmHg, measured in supine position (1). HT is the leading cause of cardiovascular morbidity and mortality worldwide (2-4). In the recent decades, prevention, detection, and treatment of hypertension have significantly reduced HT-related mortality and morbidity. However, HT and its management remain an extremely important and difficult public health problem. Primary HT is the most common form of hypertension, accounting for 90 - 95% of all cases of HT (1). Primary HT is the most prevalent cardiovascular disorder worldwide and is accompanied by significant morbidity and mortality (5). Even in developed countries, as many as 70% of the patients with primary HT suffer from badly controlled arterial Blood Pressure (BP) or complete lack of treatment (5). A critically important moment in the long process of human evolution was the adoption of upright posture. Such upright posture greatly enhanced mobility, but it created a major new burden on BP control system that had evolved mainly to meet the needs of an animal in the dorsal position. When individuals change position from supine (or sitting) to standing, a number of events occur immediately in order to maintain adequate cerebral blood flow in response to gravitational opposition. These protective changes include activation of the skeletal muscle pump and neurovascular and humoral alterations (6) along with a slight increase in SBP. When one or more of these mechanisms fail to function effectively, Orthostatic Hypotension (OH) can occur.

According to the American Autonomic Society and the American Academy of Neurology (7), OH is defined as a drop of greater than or equal to 20 mmHg in SBP or a decrease of greater than or equal to 10mmHg in DBP within 1 min of standing. OH has serious consequences, but even smaller drops in Orthostatic BP (Ortho BP) are associated with a variety of negative sequellae, such as mood difficulties (8) and impaired learning, memory, and cognition (9, 10).

## 2. Objectives

The present study aims to investigate the kinetics of the Ortho BP in primary hypertensives during the change from supine to standing position and during the standing position, using the Orthostatic Test (OT).

## 3. Patients and Methods

This prospective study was conducted on 107 primary hypertensive patients referring to the unit of exploration of the Autonomic Nervous System (ANS) in the cardiology service A at Ibn Sina Hospital in Rabat. The study was approved by the Ethics Committee of Ibn Sina Hospital after a thorough analysis. Also, written informed consents were obtained from all the patients before the tests. Each patient also completed a form recording the presence or absence of various significant functional signs.

The inclusion criteria of the study were BP equal to or higher than 140/90 mmHg measured in supine position, mild to moderate primary HT, and not having received complete treatment or not having been treated at all.

On the other hand, the exclusion criteria of the study were suffering from severe, secondary, or complicated HT and being under any anti-hypertensive treatment.

### 3.1. Orthostatic Test (OT)

OT allows the study of changes in BP and Heart Rate (HR) that occur during transition to active standing compared to supine position.

The test was performed in the morning, after fasting and under no anti-hypertensive treatment within at least 48 hours. BP and HR were monitored using a Dynamap (CRITIKON, 1846SXP) and a screen of posting (LCDCS503E, HELLIGE, EK512E), respectively. The basal systolic BP and HR were measured on both arms on a table of examination in a quiet room in supine position after a rest of at least 10 minutes. Then, OT was performed (11, 12). Ortho SBP was recorded for 10 minutes at the rhythm of 3 measurements per minute and was compared to the values of Preortho SBP. The number and gender of the patients who experienced OH were also carefully recorded.

The hypertensive patients were then classified into the following three subgroups:

-Subgroup A: Ortho SBP was higher than mean Preortho SBP by 10 mmHg or more.
-Subgroup B: Ortho SBP was lower than mean Preortho SBP by 20 mmHg or more.
-Subgroup C: -20 mmHg < (Ortho SBP – Preortho SBP) < +10 mmHg.

The changes in Ortho SBP were assessed during 10 minutes and the results were reported for each subgroup.

Peripheral sympathetic consecutive stimulation to orthostatic stress usually increases the BP. Thus, we can estimate the adrenergic peripheral alpha sympathetic nerve response according to the following formula:

Peripheral sympathetic alpha activity (in %) = [(SBPortho – SBPSP) / SBPSP] x 100

A 10 - 15% increase in adrenergic peripheral sympathetic response is considered as normal. Besides, adrenergic peripheral sympathetic response higher than 15% is normally considered as hyperactive. Finally, a sympathetic response lower than 10% is considered as sympathetic impairment (11-13).

In this study, we aim to work only on adrenergic peripheral alpha sympathetic response related to BP. HR is going to be investigated in further studies.

### 3.2. Statistical Analysis

Descriptive statistics included range, mean, and standard deviation for continuous variables and frequency and percentage for categorical ones. Categorical and normally distributed continuous variables were compared using Chi-square test and independent t-test, respectively. Additionally, the SBP changes during OT were compared using Friedman test. P values < 0.05 were considered as statistically significant. All the analyses were performed using the SPSS statistical software, version 15.0 (SPSS Inc., Chicago, II).

## 4. Results

In this study, the mean age of the patients was 55.82 ± 11.35 years ranging from 39 to 80 years. In addition, 61.7% of the participants were female.

The mean basal HR was 75.5 ± 13.2 beats/minute ranging from 65 to 87 beats/minute. In addition, the mean Preortho SBP was 150.0 ± 6.2 mmHg ranging from 147 to 163 mmHg. Besides, the mean Ortho SBP was 177.5 ± 17.3 mmHg ranging from 135 to 220 mmHg, excluding the patients with OH. The prevalence of subgroups A, B, and C was 27.1%, 15.9%, and 57.0%, respectively (Table 1).

**Table 1.** The Prevalence of Each Subgroup of Hypertensive Patients According to the Orthostatic Systolic Blood Pressure (Ortho SBP) Changes (∆SBP) During the Orthostatic Test (OT)

Sub-group	∆SBP	Number	Prevalence (%)
**A**	∆SBP ≥ +10 mmHg	29	27.1
**B**	∆SBP ≤ -20 mm Hg	17	15.9
**C**	-20 < ∆SBP < +10 mmHg	61	57.0

**Figure 1.**
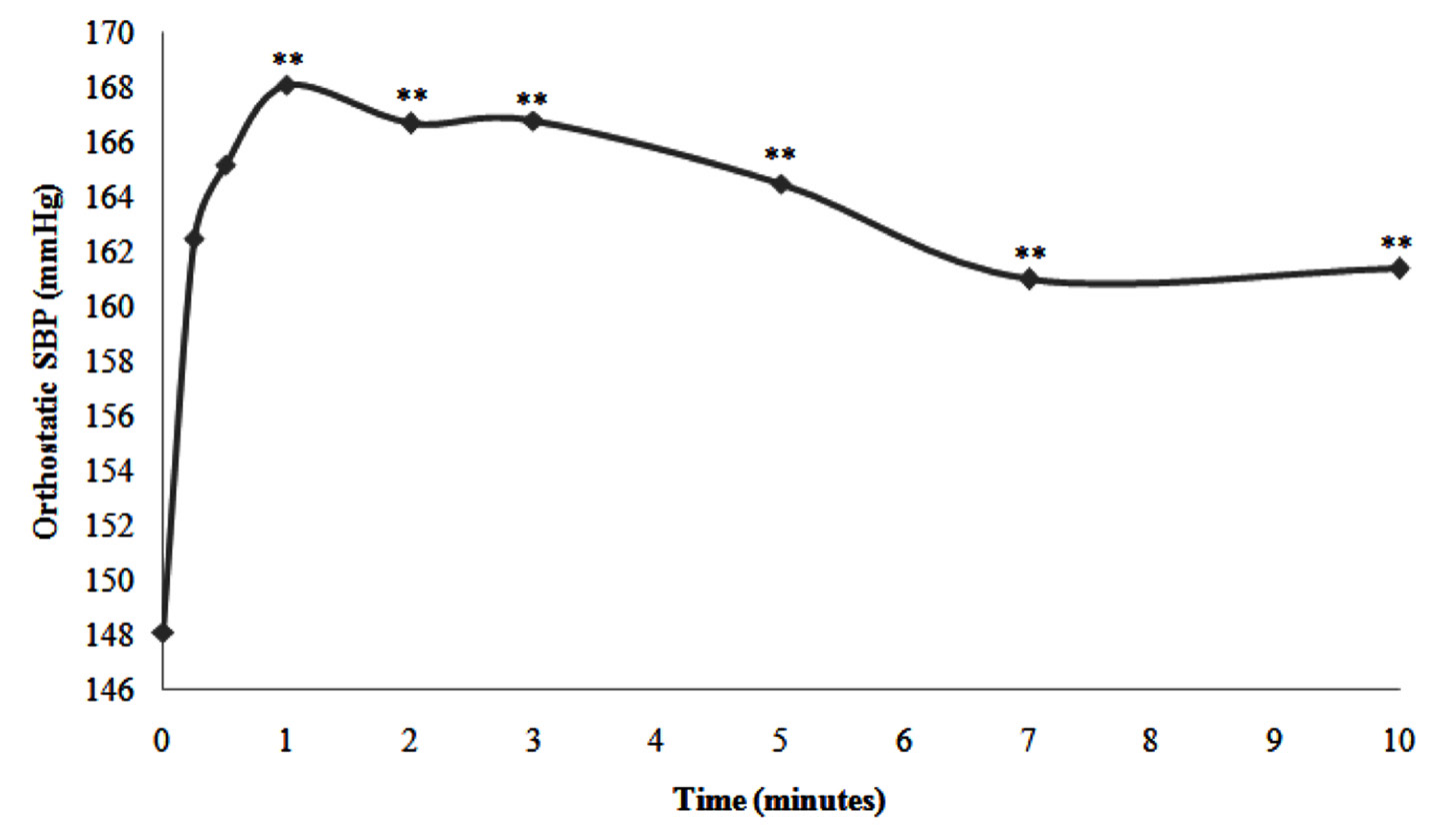
Representative Curve of the Kinetics of the Mean SBP in the Hypertensive Patients of Subgroup A during the Orthostatic Test (**: P < 0.05)

In subgroup A, 29 hypertensive patients (27.1% of the whole sample) had orthostatic HT (27.1% of total sample) out of whom, 69% (20/29) were female. The mean Preortho SBP was 148.1 ± 10.7 mmHg ranging from 141.6 to 155.2 mmHg. Besides, the mean Ortho SBP was 164.5 ± 2.7 mmHg ranging from 162 to 168 mmHg. In this subgroup, in comparison to Preortho SBP, the mean Ortho SBP increased and reached the maximum value in the first minute of OT (+20.0 mmHg) and thereafter decreased gradually throughout the OT. For example, increase in the mean ortho SBP was +16.4 mmHg in the 5^th^ minute. Even in the 10^th^ minute of the test, the increase was +13.3 mmHg. In particular, orthostatic HT was maintained (Figure 1).

**Figure 2.**
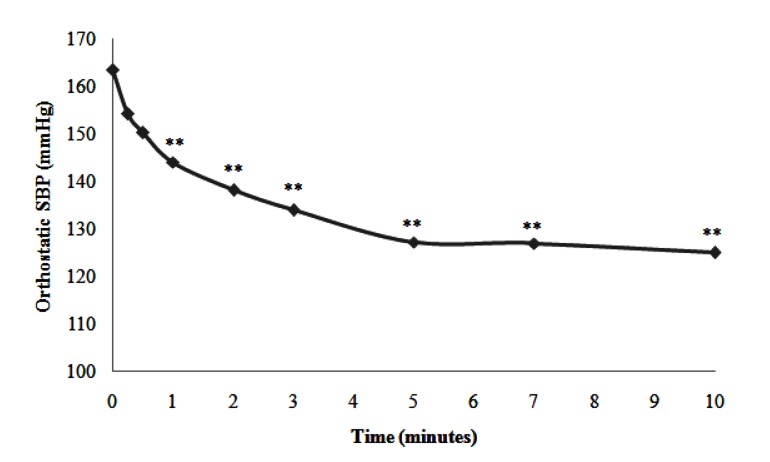
Representative Curve of the Kinetics of the Mean SBP in the Hypertensive Patients of Subgroup B during Orthostatic Test (**: P < 0.05)

In subgroup B, 17 hypertensive patients had OH (15.9% of the total population), 59% of whom (10/17) were female. The mean Preortho SBP was 163.4 ± 7.2 mmHg ranging from 160 to 167 mmHg. In addition, the mean Ortho SBP was 137.5 ± 11.2 mmHg ranging from 128 to 143 mmHg. In this subgroup, in comparison to Preortho SBP, the mean Ortho SBP decreased in the first minute of OT (-20.3 mmHg) and continued to decrease throughout the whole OT. The change was -36.2 mmHg in the 5th minute and -38.4 mmHg in the 10th minute. OH was maintained throughout the test (Figure 2).

**Figure 3.**
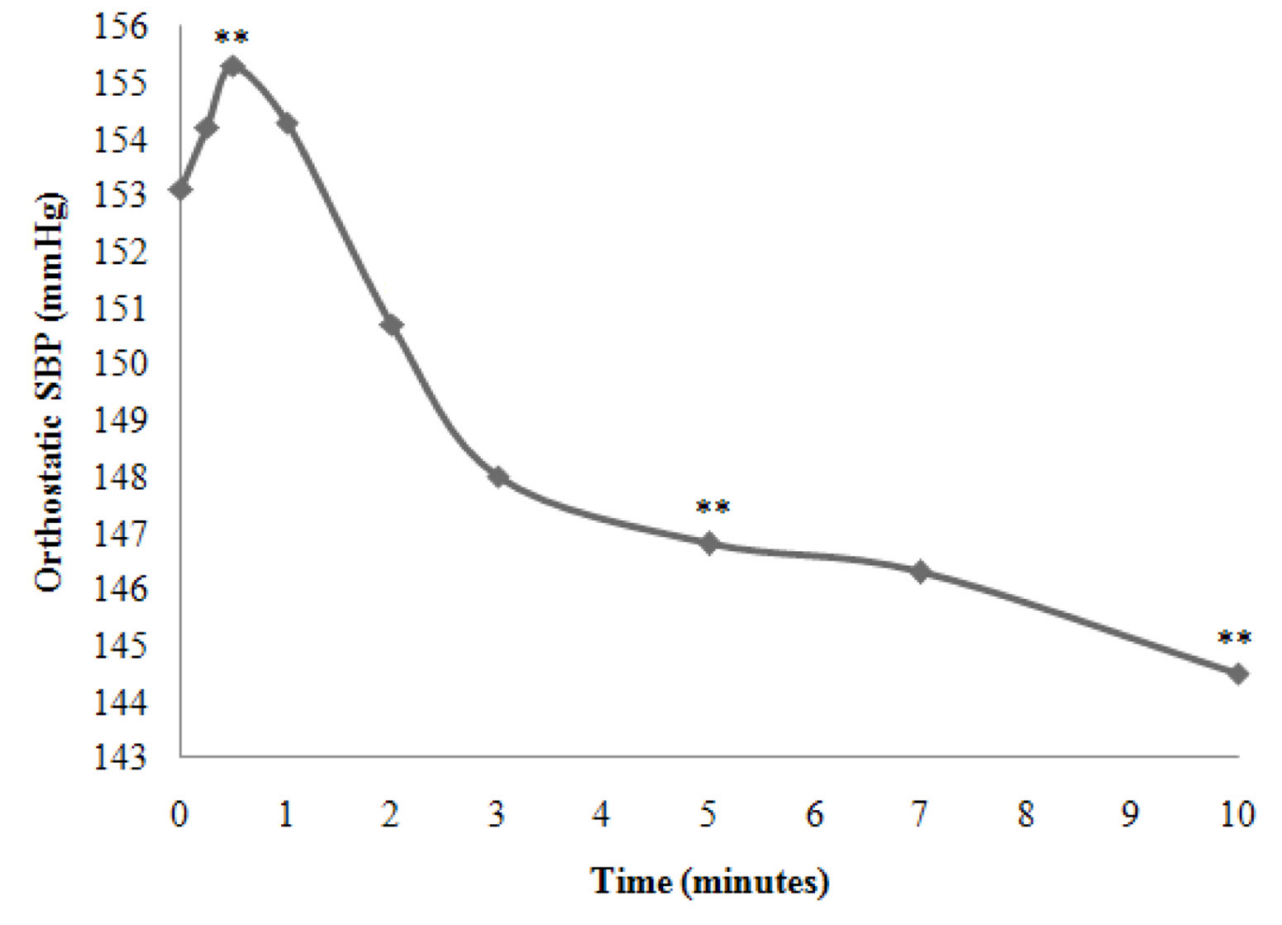
Representative Curve of the Kinetics of the Mean of the SBP in the Hypertensive Patients of Subgroup C during Orthostatic Test (**: P < 0.05)

In subgroup C, 61 hypertensive patients had no significant changes in Ortho SBP (57% of the total population), 62.3% of whom (38/61) were female. The mean Preortho SBP was 153.1 ± 8.4 mmHg ranging from 146 to 160 mmHg. Besides, the mean Ortho SBP was 150.0 ± 4.2 mmHg ranging from 145 to 156 mmHg. In this subgroup, the mean SBP increased slightly during the first minute of orthostatism (+1.67 mmHg), but decreased throughout the whole OT. The change was -5.16 mmHg in the 5th minute and -9.3 mmHg in the 10th minute (Figure 3).

Measurement of the adrenergic peripheral sympathetic alpha responses during the OT showed an average increase of 20.0%. The normal response was supposed to be around 10% in the subgroup with orthostatic HT.

## 5. Discussion

The present study aimed to investigate the kinetics of SBP in hypertensives during the change from supine to standing position and during standing position by using OT. Among the cardiovascular ANS tests, OT is a simple, non-invasive, and reproducible one involving the measurement of BP and HR during upright posture.

In this study, we reported the mean of the Ortho SBP values. Comparing Ortho SBP to the mean basal supine SPB, three subgroups of primary hypertensive patients were selected.

In subgroup A, in comparison to Preortho SBP, the mean Ortho SBP increased and reached a maximum value in the first minute of OT (+20.0 mmHg), but decreased gradually throughout the OT. In this subgroup, the orthostatic HT was maintained during the OT. Orthostatic HT refers to an increase in the BP in an upright posture. This clinical condition has been understudied and is often underappreciated in clinical practice.

In subgroup A, elevation of orthostatic BP was related to the increase in sympathetic response, as described in the literature (14-18). HT is a serious risk factor for patients. The sympathetic nerve hyperactivity and inadequate vagal response observed in hypertensives do not act solely on the cardiac system, but they also reach the other target bodies of the autonomous nervous system (19-21). The sympathetic nerve hyperactivity is regarded as a risk factor for coronaropathy, cardiac insufficiency, cerebro-vascular accident, and renal vascular attack (22). Autonomic dysregulation plays a significant role in hypertension and acts as a coronary risk factor by severe metabolic complications (23). For instance, microneurography has shown the increased activity of the sympathetic fibers specifically proportional to the severity of the essential HT, but not in secondary HT. This could help explain why some metabolic risk factors and some common essential HT diseases are not found in secondary HT (15, 24, 25).

In addition, spectral analysis techniques provide important information regarding the alterations of the nervous control of essential HT and allow highlighting an increase in sympathetic activity (15, 25).

The exact causes of sympathetic overactivity in hypertensives are only partially known. The hereditary factor has been much studied. Thus, for example, similarities found only in monozygotic twins compared to fraternal twins lead to the assumption that this factor has indeed a role in the genesis of this hyperactivity (26, 27). Besides the hereditary component, other factors may well be involved in the genesis of HT. Research in this regard has revealed autonomic imbalance complications and consequences of HT as an independent risk factor for cardiovascular diseases (26, 27).

Biochemically, measures of radiotracers of the norepinephrine passage in plasma are well suited to study the pathophysiology of increased activity of the sympathetic nervous system in HT (28). Thus, their rates are higher among the young normotensive subjects with a family history of HT compared to those with no family history of HT (29-31).

Also, same studies have found higher levels of noradrenaline in cerebrovascular circulation in the patients with primary HT compared to the healthy subjects, suggesting that an underlying increase in the central nervous system norepinephrine could form the basis of increased sympathetic impulses (15, 28).

The discovery of an autonomic imbalance in hypertensive patients not only helps to identify the mechanism of HT, but also to understand the pathophysiology of the cardiovascular system due to HT through metabolic, trophic, rheological, and hemodynamic consequences (15, 24).

Elevation of sympathetic activity increases BP, being responsible for cardiac, renal, and vascular stimulation, respectively increasing, cardiac output, fluid retention, and vascular resistance with hypertrophy of vascular smooth muscle cells (32).

Prognosis of HT depends on the reduction of sympathetic hyperactivity. It is desirable to develop antihypertensive treatment by acting as well on the sympathetic overactivity, as already mentioned by some authors (17, 33-36).

Lenski M et al. showed that Catheter-based renal denervation (RDN) reduced local and whole-body sympathetic activity and BP in the patients with resistant HT. However, safety concerns exist regarding the development of orthostatic dysfunction after RDN. In addition, RDN reduced syncopes in the patients with resistant HT three months after the treatment (37).

On the other hand, Schlaich and Krum indicated that RDN was associated with a substantial and presumably sustained BP reduction (38).

In this work, OH was found only in 17 hypertensive patients. This subgroup represented the smallest subgroup (15.9%) of the three study subgroups. However, this prevalence was not negligible. OH appears early in the OT and worsens thereafter. Masuo et al. showed that OH was present in 20% of hypertensive patients and 4% of normotensive subjects (39).

The mechanism of OH in hypertensive patients suggests impaired baroreflex function in favor of central or peripheral neuropathy affecting the afferent and/or efferent ways (39). In these situations, we observe a decrease in secretion and/or abolition of the response of catecholamines in the upright posture (40). Blomqvist demonstrated that OH was accompanied by a decrease in systolic ejection volume with an increase in adrenergic tonicity shown by increased secretion of catecholamines which is responsible for vasoconstriction (41). Masuo et al. showed that basal supine plasma Norepinephrine (NE) was greater in hypertensive patients and in the subjects with OH, while plasma NE response to upright posture was blunted in elderly hypertensive patients and in the subjects with OH regardless of HT medications (39).

When OH occurs in hypertensive patients, antihypertensive attitude becomes difficult because treatment of OH aggravates HT and vice versa. Overall, considering the severity of OH, therapeutic measures depend on the severity of OH in addition to the importance of high BP values.

Hypertensive patients with very similar supine SBP behavior can exhibit widely different ortho SBP. This has important consequences for both diagnosis and treatment of hypertensives. Overall, careful and effective treatment of hypertensives requires careful consideration and assessment of orthostatic BP.
